# Extracellular Vesicles and the Oviduct Function

**DOI:** 10.3390/ijms21218280

**Published:** 2020-11-05

**Authors:** Emily A. Harris, Kalli K. Stephens, Wipawee Winuthayanon

**Affiliations:** Center for Reproductive Biology, School of Molecular Biosciences, College of Veterinary Medicine, Washington State University, Pullman, WA 99164, USA; emily.harris3@wsu.edu (E.A.H.); kalli.stephens@wsu.edu (K.K.S.)

**Keywords:** egg, embryo, extracellular vesicle, exosome, fallopian tube, microvesicle, oocyte, oviductosome, oviduct, sperm

## Abstract

In mammals, the oviduct (or the Fallopian tube in humans) can be divided into the infundibulum (responsible for oocyte pick-up), ampulla (site of fertilization), isthmus (where preimplantation embryos develop), and uterotubal junction (where embryos transit to the uterus). The oviductal fluid, as well as extracellular vesicles produced from the oviduct epithelial cells, referred to as oEVs, have been shown to improve the fertilization process, prevent polyspermy, and aid in embryo development. oEVs contain molecular cargos (such as miRNAs, mRNAs, proteins, and lipids) that can be delivered and fuse to recipient cells. oEVs produced from the ampulla appear to be functionally distinct from those produced from the isthmus. In multiple species including mice, cats, dogs, pigs, and cows, oEVs can be incorporated into the oocytes, sperm, and embryos. In this review, we show the positive impact of oEVs on gamete function as well as blastocyst development and how they may improve embryo quality in in vitro conditions in an assisted reproductive technology setting for rodents, domestic animals, farm animals, and humans.

## 1. Introduction

Extracellular vesicles (EVs) participate in intercellular and interorganismal communications (reviewed by [1]). EVs is the collective term used for both exosomes and microvesicles. Exosomes are vesicles with an approximate size of 30–100 nm in diameter. Exosomes are derived from endocytotic origin and are released from cells through plasma membrane fusion of a multi-vesicular body [2]. However, microvesicles (100–1000 nm) readily bud from the cell membrane (reviewed by [3]). EVs communicate their signal to recipient cells by transferring their molecular cargos using endocytosis and cellular fusion [4]. These cargos usually contain DNA, RNA, proteins, and other metabolites [1]. In this review, we have described characteristics of EVs in the oviduct from different species and their role in supporting oocytes, sperm, and embryos.

## 2. Extracellular Vesicles

### 2.1. General Characteristics of EVs

Exosomes are formed as intraluminal vesicles (ILVs) within multivesicular bodies (MVBs). MVBs then either fuse with cellular lysosomes and degrade, or fuse with the plasma membrane. MVB-fused plasma membranes are released as exosomes into the extracellular space (reviewed by [5]). Specific markers for exosomes used in different studies of oviductal EVs (oEVs) have also been identified. These oviduct exosomal markers include tetraspanin transmembrane superfamily (i.e., CD9, CD63, and CD81), heat shock proteins (HSPs; i.e., HSPA1A, HSP70, and HSPA8), annexin A1 (ANXA1), actin-linking ezrin-radixin-moesin (ERM), and tumor susceptibility gene 101 (TSG101) [6].

In contrast to exosomes, microvesicles are formed by an outward budding from the plasma membrane [7]. Marker proteins for microvesicles include TSG101, arrestin domain containing 1 (ARRDC1), gelatinases, ADP ribosylation factor 6 (ARF6), major histocompatibility complex 1 (MHC-1), β1-integrin, vesicle-associated membrane protein 3 (VAMP3), and membrane type 1-matrix metalloproteinase (MT1MMP) [8]. Apoptotic bodies are also considered a subclass of EVs. These apoptotic bodies are released only during apoptotic cell death and have molecular signals that attract phagocytes and promote apoptotic cell clearance (reviewed by [9]). However, we will focus solely on exosomes and microvesicles in this review.

### 2.2. EV Biogenesis

Proteins involved in the biogenesis of exosomes include Ras-related proteins (RAB11/RAB35, RAB27A/B, RAB7), diacylglycerol kinase alpha (DGKα), and vesicle associated membrane protein 7 (VAMP7) [8]. *Rab11a*^−/−^, *Rab35*^−/−^, and *Rab7*^−/−^ mice are embryonically lethal in mice [10,11,12]. Rab27a/b double knockout mice have shown deficiency in exosome secretion, leading to a low-grade inflammatory phenotype [13]. However, *Dgkα*^−/−^ and *Vamp*7^−/−^ mice are viable [14,15]. Proteins involved in the biogenesis of microvesicles include ARRDC1, TSG101, vesicle-fusing ATPase (VSP4), RAB22A, hypoxia-inducible factors (HIF), ARF6, phospholipase D (PLD), extracellular-signal-regulated kinase (ERK), and myosin light-chain kinase (MLCK) [8]. However, only TSG101 and ARF6 appear to have indispensable biological functions in mammals as *Tsg101*^−/−^ mice die around the time of implantation [16] whereas *Arf6*^−/−^ mice die during mid-late gestation [17]. These findings indicate that some of the proteins involved in EV biogenesis are crucial for cellular function in mammals.

## 3. Characteristics and Regulation of EV Biogenesis in the Oviduct

### 3.1. Characteristics of Oviductal EVs in Different Species

**Figure 1:**
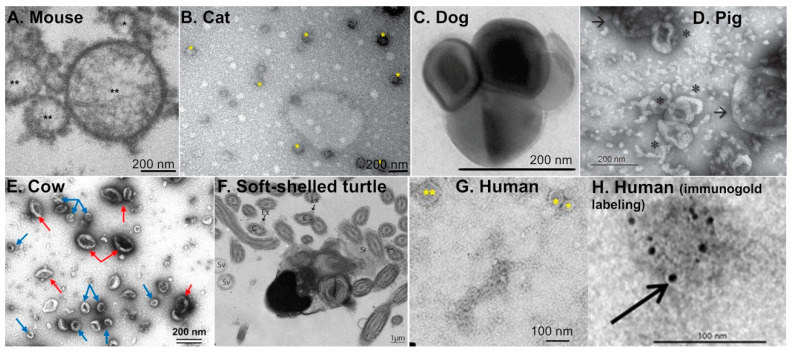
Transmission electron microscopy images of extracellular vesicles present in the oviductal fluid from different species, including rodents, domestic animals, farm animals, reptiles, and humans. (**A**) Mouse: exosome (*) and microvesicles (**). Reprint with permission from [23]. (**B**) Cat: extracellular vesicles (*). Reprint with permission from [28]. (**C**) Dog: oEVs. Reprint with permission from [25]. (**D**) Pig: exosomes (*) and microvesicles (arrows). Reprint with permission from [26]. (**E**) Cow: exosomes (blue arrows) and microvesicles (red arrows) isolated from in vivo oviduct. Reprint with permission from [24]. (**F**) Soft-shelled turtle: extracellular vesicles (arrows) in the lumen of the oviduct from the isthmus region. Reprint with permission from [27]. C; cilia, Ex; exosome, Sr; oviduct secretions, Sv; secretory vesicles. (**G**,**H**) Human: exosomes (*) and microvesicles (**) with or without immunogold labeling. Reprint with permission from [19].

Within the past decade, extracellular vesicles of the oviduct (or oEVs) have been studied in various species including rodents, domestic and farm animals, reptiles, as well as humans (Figure 1A–H) [6]. However, most studies characterizing oEVs have been performed using cows and pigs. Almiñana and Bauersachs recently provided a comprehensive review on the current understanding of biomolecular contents in the oEVs present in various species, which include miRNAs, small non-coding RNAs (ncRNAs), RNAs, and proteins [6,18]. Biogenesis of oEVs appears to be via the apocrine pathway [19], in which EV contents are released into the luminal fluid by pinching off of the plasma membrane. This apocrine pathway was also observed in EVs found in the epididymis [20,21]. Studies showed that CD109 can be used as a marker for oEVs as it was highly abundant in both porcine and bovine oviductal fluid [22]. As mentioned earlier, there are three major types of EVs: exosomes, microvesicles, and apoptotic bodies. The diameter for EVs found in the oviductal fluid varies between species (Table 1) and ranges from 30 to 150 nm for exosomes, >100–1000 nm for microvesicles, and 1–5 µm for apoptotic bodies. Based on the size distribution, oEVs from all species studies are mostly exosomes. Nevertheless, there is some overlap in the size of EVs depending on the species and characterization methods, which will be discussed later in this review.

In humans, exosomes are less than 100 nm and microvesicles are 0.1–1 µm in diameter [19]. In cows, exosomes are 30–100 nm in diameter whereas microvesicles are 100–250 nm in diameter [24]. Alcântara-Neto and colleagues established that exosomes are 30–150 nm in diameter whereas microvesicles are greater than 150 nm in pigs. In addition, the ratio of exosomes to microvesicles in the oviductal fluid of pigs is 83% to 17%, respectively [26]. In mice, oviductal exosomes are 25–100 nm in diameter [29]. However, exosomes were not distinguished from microvesicles in this study, but were referred to as “oviductosomes” [29]. Extracellular vesicles are approximately 40–150 nm in diameter in cats, [28] and 50–130 nm in turtles [27]. Ferraz and colleagues showed that oEVs from dogs are approximately 158.9 nm in diameter [25]. Based on this study, the concentration of oEVs in the domestic cat ranges from 2.0 × 10^10^ to 4.5 × 10^10^ particles/mL and 1.8 × 10^10^ to 1.8 × 10^11^ particles/mL in the domestic dog [25]. In oviductal fluid isolated from cats, exosomes are the most prevalent type of EVs. oEVs from domestic cats contain approximately 1,511 proteins, which is more than three-times the number of proteins identified in bovine oEVs [28]. Note that the different techniques used for the characterization of oEVs may cause an artifact in size measurement [30]. Although the size of oEVs appears to be different in each species, EVs are present in the oviduct of both mammalian and reptilian species (Figure 1).

Protein cargos in EVs appears to be more common between cows and humans (171 proteins in common), compared to sheep (16 proteins in common) [22,31,32]. Proteins found in common between bovine oEVs and human endometrial EVs include proteins that are related to exosome biogenesis (endosomal sorting complexes required for transport (ESCRT)-associated proteins), intracellular vesicular trafficking (cytoplasmic dynein 1 light intermediate chain 1 (DYN-C1LI1), synaptosomal-associated protein 23 (SNAP23), and syntaxin-binding protein 1 (STXBP1)). This may suggest that oEV protein cargo is more conserved between cows and humans than between cows and sheep. However, Almiñana and colleagues pointed out that the method of hormone induction of the protein cargo in these individual studies may have also contributed to differences and variability of cargo content (see details on hormonal regulation of oEVs below).

### 3.2. Region- and Cell-Specific Regulation of oEVs Biogenesis

In mammalian species, the oviduct is divided into four regions: the infundibulum (which is responsible for oocyte pick-up after ovulation), the ampulla (the site of fertilization), the isthmus (where the majority of preimplantation embryonic development occurs), and the utero-tubal junction (where embryos transition to the uterus). Lopera-Vasquez and colleagues showed that addition of bovine oEVs collected from the isthmus, but not the ampulla, in embryo culture significantly increased the development of embryos to the blastocyst stage [7]. However, the oEVs from the ampulla, but less so from the isthmus, increases sperm intracellular Ca^2+^ which aids in capacitation [33]. Furthermore, oEVs vary in size depending on which region of the oviduct they are obtained from. In the ampulla, oEVs are approximately 35 and 190 nm in diameter, compared to 25, 150, and 400 nm oEVs found in the isthmus [33]. Ampullary oEVs have 3–4 fold higher total protein concentration compared to those from the isthmus [33]. Supplementation with oEVs collected from the ampulla and isthmus improved bovine sperm viability in vitro [33]. Moreover, oEVs collected from the bovine isthmus increased blastocyst formation at a higher rate when compared to oEVs collected from the ampulla [7] (see detail discussion in later section). These studies indicate that oEVs produced from different regions of the oviduct affect their molecular cargos and physiological functions on recipient cells. Waqas and colleagues demonstrated that ciliated cells produced and secreted EVs into the oviduct of turtles [27]. As ciliated cells are concentrated at the ampulla, whereas secretory cells are mainly abundant in the isthmus [34], it is possible that ciliated and secretory cells differentially produce distinct extracellular vesicles. It is also possible that secretory cells in the ampulla may differentially secrete oEVs than the secretory cells in the isthmus region. Therefore, the spatiotemporal regulation of oEVs from different oviductal regions may provide unique biological actions that are tailored for sperm migration/function, fertilization, preimplantation embryo development, and embryo transport.

### 3.3. Differences between In Vivo- vs. In Vitro-Derived oEVs

Although the size distribution of oEVs produced from either in vivo or in vitro are relatively similar, protein cargo differs between in vivo- vs. in vitro-derived oEVs [24]. Specifically, Almiñana and colleagues used mass spectrometry analysis to identify 175 proteins that are common between in vivo- vs. in vitro derived oEVs [24]. Fewer proteins were identified in in vitro-derived oEVs (222) compared to in vivo-derived oEVs (270). There are 97 proteins unique to in vivo-derived oEVs while 47 proteins are exclusively present in in vitro-derived oEVs [24]. For example, CD109 antigen is present only in in vivo-derived oEVs whereas lactadherin (or MFGE8), a glycoprotein involved in adhesion, fertilization, and the clearance of apoptotic cells [35], is only found in in vitro-derived oEVs. Myosin heavy chain 9 (MYH9) and HSPA8 are produced both in vivo and in bovine oviduct epithelial cell primary culture (bOECs). However, another oviductal glycoprotein (OVGP1, encoded by mucin 9 or *MUC9*) is only present in oEVs isolated from in vivo, not from cultured bOECs [24]. Importantly, the presence of OVGP1 has been shown to improve preimplantation embryo development [36], suggesting that these differences in cargo content from in vivo- vs. in vitro-derived oEVs may contribute to the discrepancy between the effect of different sources of oEVs on preimplantation embryo development. Proteins related to oEV biogenesis including tetraspanins and RAB family proteins are commonly present in oEVs collected from both in vitro and in vivo. Most annexin proteins, which have roles in membrane scaffolding, trafficking, fertilization, and reducing inflammation, were identified in in vitro-derived oEVs, except ANXA7 which is only present in in vivo-derived oEVs [24]. Recently, a 3D culture of oviduct epithelial cells showed that oEV content is similar to that of in vivo-derived oEVs, compared to those of monolayer culture of epithelial cells [37,38]. These data suggest that differences in protein cargo content between in vivo vs. in vitro are likely due to a lack of paracrine regulation from other cell types in the in vitro monoculture settings.

### 3.4. Hormonal Regulation of oEVs

It is well-established that the histoarchitecture and biochemical properties of epithelial cells in the female reproductive tract are affected by ovarian steroid hormones, including estradiol (E_2_) and progesterone (P_4_). The volume of oviductal fluid has shown to increase during the estrus stage in the ovine oviduct [39,40]. Furthermore, the temperature of the oviductal fluid differs across the estrous cycle [41]. These data suggest that E_2_ and P_4_ regulate the production of oviductal fluid and environment within the oviduct. As oEVs are cellular products, changes in epithelial cell morphology could alter content and biological functions of oEVs. There are numerous studies regarding hormonal regulation of EVs in the uterus in several species. However, few studies focus on the oviduct. In cows, there were no differences in oEV size or concentration across the estrous cycle [22]. However, differences in ncRNAs, micro-RNAs (miRNAs), as well as protein composition were observed in oEVs isolated at different stages of the estrous cycle [22], suggesting that ovarian steroid hormones may regulate oEV production and secretion.

In cows, mRNA and protein content in the oEVs during stage 1 (S1; ovulated follicle; days 1–4 of the estrous cycle) are distinct from those at S2 (early luteal phase; days 5–11), S3 (functional corpus lutea (CL); days 11–17), and S4 (regressing CL with large preovulatory follicles; days 18–20) [22]. Of these differences, *HSPA8* is expressed at higher levels, whereas *MYH9* is expressed at lower levels during S1 compared to other stages [22]. However, *CD109* is not differentially expressed across the four stages. Additionally, many proteins identified in oEVs related to embryo development and gamete interactions are differentially expressed across the four stages suggesting a role in hormone regulation of EVs to support fertilization and preimplantation embryonic development. Abundant proteins isolated from oEVs during S1 are related to ribosome biogenesis, protein translation, folding, transport and processing, and focal adhesion as identified by GO terms [22]. Proteins from oEVs during S2 are associated with ribosomes, protein translation and transport, and focal adhesion. S3 showed an increase in proteins involved in the enrichment for fructose/lipid/GDP binding, cytoskeleton organization and cell motility, cytoplasmic vesicles, signal transduction, and secretory processes. Because S3 correlates with an increase in circulating P_4_, this suggests that these identified GO processes, including secretory action in the oviduct, are potentially regulated by P_4_. Therefore, P_4_ is likely an important regulator of the oviductal fluid protein content that is specific to cell signaling and pregnancy establishment. Proteins involved in the aforementioned GO processes were downregulated during S4 when P_4_ levels are low, due to CL regression, further indicating that P_4_ is an important regulator of oviductal protein content for pregnancy establishment. This study provides biological insights as it examined hormonal regulation of oEVs isolated directly from cycling animals rather than inducing changes in vitro via artificial supplementation of E_2_ or P_4_. As mentioned previously, the contents of the EVs differ depending on their in vivo or in vitro origin [24]; it is very likely that these differences in protein content are under the influence of steroid hormones. As such, a lack of appropriate hormonal regulation, in addition to paracrine cell signaling, is missing in the in vitro culture condition.

It is challenging to differentiate whether proteins measured in the oviductal fluid derive from the EVs. Insights from studies of protein content in the oviductal fluid could support findings in oEVs. Hormone levels vary across the estrous cycle and affect the protein content in oEVs, suggesting that E_2_ and P_4_ likely modulate oEVs in the oviductal fluid. It is possible that local secretion of hormones from either side of the oviduct may affect oEV production and oviductal fluid global protein content. Lamy and colleagues showed that P_4_ levels in the bovine oviductal fluid varied depending on the side of ovulation as well as the stage of the estrous cycle [42]. The highest P_4_ levels were during the mid-luteal phase and the ipsilateral side (same side of the oviduct as ovulation) was positively correlated with increased levels of P_4_, which were 10–50 times the concentration of P_4_ in circulation. E_2_ concentration, however, was not dependent on the side of ovulation, but depended more on the stage of the estrous cycle, with the highest levels during the pre-ovulatory phase. In addition, P_4_ and E_2_ concentrations in oviductal fluid were found to be 2–4 times less than P_4_ and E_2_ concentrations in the oviductal tissue [42].

Protein analysis showed that 482 proteins were identified in the oviductal fluid during the estrous cycle, and 82% of these proteins were common between the ipsilateral and contralateral sides [43]. The most highly expressed proteins in the ipsilateral post-ovulatory fluid were HSPs including HSPA8, HSP90, HSPA5 (a 78-kDa glucose-regulated protein or GRP78), and HSPA6. HSPs are among the most abundant proteins in bovine oEVs [22] and are among the first proteins produced during fertilization and preimplantation embryo development [44]. These data suggest that HSPs may be produced in the ipsilateral oviduct, as opposed to the contralateral oviduct, in response to the presence of gametes and subsequently facilitate embryo development. The most highly expressed protein in ipsilateral pre-ovulatory oviductal fluid was ANXA1, which could also play a role in preparing the ipsilateral oviduct for sperm interactions.

The most abundant proteins in ipsilateral oviductal fluid overall (unspecific to pre- or post-ovulation) were CD109, CCT5, CCT8, and CCT6A. Post-ovulatory fluid contained higher levels of HSPs including GRP78, HSPA8, HSPA6, HSP90 AA1, AB1, and B1 compared to the mid- and late-luteal phase, once again suggesting their role in fertilization and early embryo development. Pre-ovulatory fluid contained higher levels of phosphatidylethanolamine-binding protein 1 (PEBP1), CD109 and proxiredoxin-2 (PRDX2). PEBP1 has been implicated as a decapacitation factor [45,46] and therefore likely participates in the regulation of gamete interactions. OVGP1 was more highly expressed during the pre and post-ovulatory phase compared to the mid-luteal phase whereas thioredoxin (TXN) was more highly expressed during the pre-ovulatory phase compared to the post-ovulatory or mid-luteal phase. A positive correlation was identified between P_4_ levels and PEBP1, PRDX2, β-actin (ACTB), high mobility group protein B1 (HMGB1) and ribosomal protein L18 (RPL18), whereas a negative correlation between P_4_ and septin-9 (SEPT9) and ribosomal protein S 19 (RPS19) was observed [43]. These data suggest that levels of P_4_ and E_2_ in the oviductal tissue may contribute to differential secretion and production of oEVs as well as regulation of oEV cargo content that may be important for fertilization and embryo development. Because fertilization and embryo development occur in the ipsilateral oviduct, these data provide information about the difference in microenvironment between the ipsilateral and contralateral oviduct that may contribute to successful pregnancy establishment.

Previous studies have shown that glycosidases in bovine oviductal fluid have the ability to modify the zona pellucida (ZP), thereby affecting sperm binding and fertilizing capacity [47]. Furthermore, glycosidase activity has been shown to vary across the estrous cycle [48,49] suggesting that steroid hormone levels regulate glycosidase activity in the oviductal fluid and may contribute to fertilizing capacity of the sperm. Carrasco and colleagues demonstrated that, α-l-fucosidase, β-*N*-acetyl-glucosaminidase, β-d-galactosidase, α-d-mannosidase and β-*N*-acetyl-galactosaminidase activity were present in bovine oviductal fluid [48]. However, α-d-mannosidase and β-*N*-acetyl-galactosaminidase activity was elevated during the follicular phase [48]. Moreover, α-d-mannosidase, β-*N*-acetyl-galactosaminidase, and β-d-Galactosidase activity were also increased during the late follicular phase in porcine oviducts [49]. These data implicate E_2_ levels in being a positive regulator of glycosidase activity in bovine and porcine oviductal fluid.

In pigs, oEV concentration, size, and protein content change across the estrous cycle [50]. This is different to cows as there are no observable differences in the concentration or size distribution across the estrous cycle [22]. In pigs, the number of oEVs collected during the post-ovulatory stage, when P_4_ starts to rise, was significantly larger compared to estrus (when E_2_ and LH levels peak), late estrus, and diestrus. Similarly, the protein concentration of these oEVs was also significantly higher in oviductal fluid collected during the post-ovulatory phase compared to estrus, late estrus, and diestrus. However, these oEVs were smaller when the oviductal fluid was collected from the post-ovulatory group compared to estrus and late estrus, unlike that of bovine oEVs, suggesting a species-specific hormonal regulation of oEV size. Flotillin-1 (FLOT1) and FLOT2, vesicular trafficking proteins, are most abundant during the post-ovulatory stage, suggesting P_4_-dependent regulation. Olfactomedin 3 (OLFM3), a scaffolding protein that has been implicated in embryo development [51], gradually decreased in oEVs isolated from estrus and late estrus. However, OLFM3 was absent in oEVs isolated from post-ovulatory stage. Talin-1 (TLN1), a cytoskeletal protein, levels are the lowest during the post-ovulatory stage [50]. This would suggest that OLFM3 and TLN-1 expression is stimulated by E_2_ and/or suppressed by P_4_. Contrary to other studies, MYH9 and CLTC (clathrin heavy chain) were most abundant during the pre-ovulatory phase rather than the post-ovulatory phase [50]. These data may suggest differences in oEV protein content among species.

In mice, plasma membrane calcium-transporting ATPase proteins (PMCA4 and PMCA1) were detected in oEVs and vary across the estrous cycle [19]. PMCA1 was highly expressed in murine oEVs during estrus, but not in uterine EVs, indicating that protein cargo, especially PMCAs, is specific to oviductal tissues. These data suggest a role for hormonal regulation of PMCA proteins during fertilization and preimplantation embryonic development. Specific functions of PMCA proteins are discussed in later sections below.

Because obtaining human oEVs/oviductal fluid is not feasible, especially during early pregnancy, we are limited in our ability to understand oviductal fluid protein and the role of steroid hormones in regulated oEV protein content and how it supports pregnancy establishment in humans. As mammary tissues are hormonal target organs, studies of EV in human mammary cells could provide useful insights into how steroid hormones regulate oEV protein content in human Fallopian tubes. In cultured human mammary epithelial cells, E_2_ treatment led to the secretion of smaller exosomes and increased protein expression levels of exosome markers such as CD63 and FLOT1, CD133, ANXA2, catalase, and high molecular weighted ubiquitinated proteins [52]. This study indicates that E_2_ impacts EV secretion and protein content in human cells. This phenomenon of hormone regulation of EVs in mammary cells may correspond to Fallopian tube biology as well, but further exploration is required to address this knowledge gap.

Together, these findings show that hormonal regulation of oEVs remains an area that requires much more investigation to gain a better understanding of their role in fertilization, preimplantation embryonic development, and embryo transport. Furthermore, more research of mammalian oviductal fluid and oEVs is needed to understand its fundamental roles in reproductive function in vivo.

## 4. Biological Functions of oEVs for Gametes and Embryos

### 4.1. Effect of oEVs on Oocytes

As ovulated oocytes are bathed in both follicular fluid and oviductal fluid, it is difficult to distinguish the sources and their effect of EVs on oocyte function. In this review, we only emphasize the literature with findings specifically on EVs from the oviductal fluid on oocyte maturation. Following in vitro fertilization (IVF), oocytes that were matured in vitro showed decreased rates of blastocyst formation compared to oocytes matured in vivo [53]. Lloyd and colleagues demonstrated that embryos, from oocytes treated with oviductal fluid, were more likely to develop to the blastocyst stage; compared to those without [54]. It was proposed that the oviductal fluid provides the embryo with protection against apoptosis and adverse regulation of mitochondrial DNA [54].

**Figure 2:**
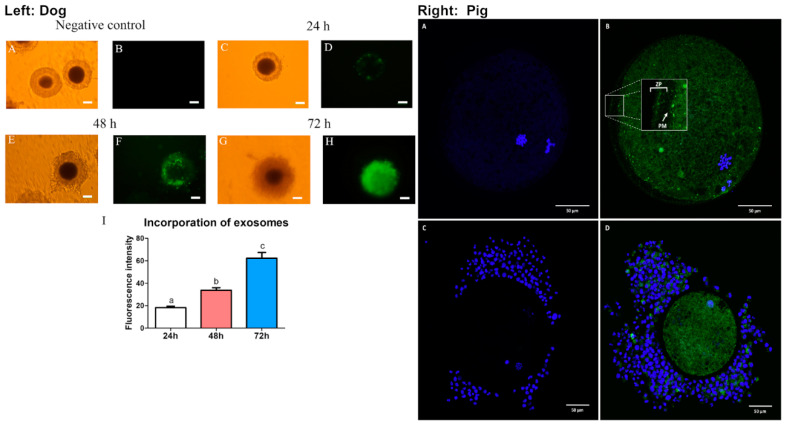
EVs from the oviduct are transferred to the oocytes in dogs (left panel) and pigs (right panel). (**Left**) Canine oEV incorporation in the canine cumulus-oocyte increases over time. (**A**,**B**) negative control, (**C**,**D**) 24 h, (**E**,**F**) 48 h, and (**G**,**H**) 72 h after incubation with (PKH67)-labeled canine oEVs. (**I**) Incorporation of exosomes as measured by fluorescent intensity over time. Bright field images (**A**,**C**,**E**,**G**), green fluorescent (PKH67) images (**B**,**D**,**F**,**H**). Length of scale bars was not indicated in the original study. Reprint with permission from [58]. (**Right**) Images of oocytes from pigs after 20 h of incubation in the presence of (**A**,**C**) negative control or (**B**,**D**) 0.4 μg/μL of green florescent (PKH67)-labelled porcine oEVs. (**A**,**B**) porcine denuded mature oocytes. (**C**,**D**) cumulus-oocyte complex. ZP; zona pellucida, PM; plasma membrane, blue; Hoechst staining, green; PKH67-EVs staining. Scale bars; 50 μm. Reprint with permission from [59].

Studies in dogs showed that the incubation of oviductal microvesicles in combination with oviduct epithelial co-culture was optimal for metaphase II (MII) oocyte maturation [55]. Microvesicles at the concentration of 75–100 × 10^6^/mL promoted the highest MII oocyte maturation rate (20.34–21.82%) compared to 8.66% in control (no addition of microvesicles nor co-culturing with oviduct epithelial cells) or to 10.71% when cultured with the epithelial cells alone. However, the co-culture of oocytes with multicellular spheroids yielded 20% of oocyte maturation [55]. This suggests that multicellular spheroids better mimic what occurs in vivo compared to the monolayer of epithelial cells and that these oviduct epithelial cells secrete important factors in microvesicles that interact with the oocyte in vivo. Nevertheless, incubation of oocytes with higher concentrations (150 × 10^6^/mL) of microvesicles caused toxicity, resulting in lower maturation rates (9.26%). Additionally, Lange-Consiglio and colleagues showed that the cargo content in microvesicles isolated from canine oviducts contained miRNAs that are important for follicular growth and oocyte maturation, including miR-30b, miR-375, and miR-503 [55]. These miRNAs have been shown to target several pathways that are important for oocyte viability and early embryonic development including WNT, MAPK, and TGFβ. As the study showed that higher concentrations of MVs resulted in lower maturation rates, it was hypothesized that high levels of miR-375 may cause a toxic effect in the culture media. Accordingly, miR-375 has been shown to inhibit the TGFβ pathway, thereby disrupting oocyte maturation in mares [56,57]. Lastly, microvesicles were incorporated into the cumulus cells after 48 h and were visibly present in the oocyte cytoplasm by 72 h (Figure 2). In agreement with this finding, exosomes from the canine oviduct can be incorporated into cumulus cells and cytoplasmic compartment of the oocytes (Figure 2) [58]. In addition, the incubation of exosomes from oviduct epithelial cells increased oocyte maturation rate to the MII phase (22.5% compared to 9% in the group without exosomes) in dogs [58]. This effect of canine exosomes on oocyte maturation appeared to be acting through the activation of the EGFR/MAPK signaling pathway [58].

In addition to dog oEVs, pigs’ oEVs were also shown to be incorporated into the oocytes with or without the presence of the cumulus cells (Figure 2) [59]. Moreover, incubation of bovine and porcine oocytes with oviductal fluid also improved resistance against proteolytic degradation as well as decreased ZP binding thereby reducing polyspermy [26,59] (discussed in more detail in the next section). These studies indicate that both exosomes and microvesicles present in the oviductal fluid have positive impacts on oocyte maturation as well as ZP functionality during fertilization. Although oEVs have been shown to improve oocyte maturation in dogs, it is unclear whether these oocytes would therefore have improved fertilization rate and give rise to higher quality embryos. More studies are needed to evaluate the effects of oEVs on oocyte quality and function in other species and how the quality of oocyte maturation in the presence of oEVs affect downstream embryo development.

### 4.2. Effect of oEVs on Sperm

There have been numerous studies focusing on the oviductal fluid and how it regulates sperm function and polyspermy, especially in farm animals. Within the past decade, studies have shifted focus to the influences of oEVs on sperm function.

**Figure 3:**
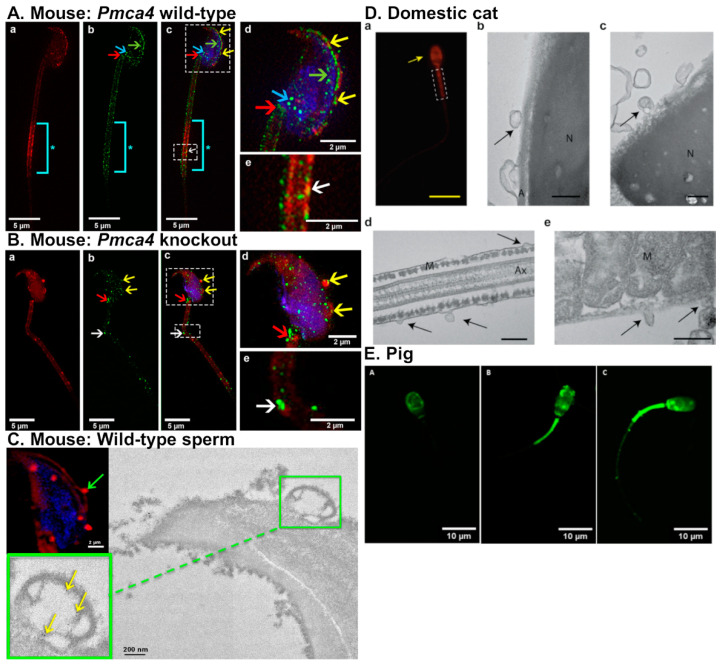
EVs from the oviduct are transferred to the sperm using fusogenic mechanism. (**A**–**C**) Mouse sperm. (**A**) *Pmca4*^+/+^ and (**B**) *Pmca4*^−/−^ sperm incubated with FM4–64FX-labeled oviductosome (red fluorescent signal) and PMCA4 antibody (green fluorescent signal). Yellow arrows; acrosome, white arrows; sperm midpiece, blue arrows; posterior head, red arrows; neck, *; distal midpiece; aqua bar, intense staining of FM4–64FX-labeled oviductosomes. Sperm nuclei were stained with DRAQ5 (blue). (**C**) Fusion of microvesicles on the sperm membrane from WT mice. Green arrow; fusion of FM4–64FX-labeled microvesicles over the acrosome. Transmission electron microscopy (TEM) analysis indicates the fusion (green arrow). Inset shows transfer of gold particles (PMCA4, yellow arrow) from OVS to the sperm membrane over the acrosome. Reprint with permission from [23]. (**D**) Uptake of oEVs by sperm in domestic cats: (a) cat sperm incubated with red-fluorescent labeled oEVs, arrow indicates that EVs bind to sperm acrosome and mid-piece (dashed box). TEM images (b–e) of sperm incubated with EVs (black arrows), indicating that EVs bound to sperm head (b,c) and mid-piece (d,e). Abbreviations: N; nucleus, A; acrosome, Ax; axoneme, and M; mitochondria. Scale bar: yellow; 15 μm, black; 200 nm. Reprint with permission from [28]. (**E**) Incorporation of porcine oviductal EVs labeled with PKH67 (a green fluorescent dye) at different regions of pig sperm including (A) head, (B) head and intermediate piece, and (C) head, intermediate piece, and flagella. Reprint with permission from [59].

In mice, oEVs were firstly termed “oviductosomes” by Al-Dossary and colleagues [29]. It was demonstrated that PMCA4, a Ca^2+^ efflux pump essential for male fertility, is secreted by murine oviduct epithelial cells in oEVs and interacts with sperm [29]. Moreover, PMCA4 regulates Ca^2+^ levels and therefore promotes sperm hyperactivation and the acrosomal reaction. PMCA4 is up taken by the sperm acrosome, head, midpiece, and principal piece, in that order (Figure 3A), via fusion proteins, αvβ3 integrin (αv subunit) and CD9 [23,29]. Incubation of fluorescent-labeled oviductosomes are incorporated into *Pmca4*^−/−^ sperm at the acrosomal region as well as neck and midpiece (Figure 3B). PMCA4 can be delivered to sperm by both exosomes and microvesicles via a fusogenic mechanism (Figure 3C) [23]. In the absence of PMCA4, male mice are infertile due to inefficient sperm hyperactivation and motility [60,61]. Female *Pmca4*^−/−^ mice, however, have normal fertility. Additionally, sperm PMCA4 increases in the presence of oviductal fluid and oviductosomes [29].

Bathala and colleagues detected PMCA4 in oEVs from the luminal fluid collected from Fallopian tubes in women undergoing hysterectomies [19]. This finding suggests that PMCA4 may have a role in human sperm function and it is possible that PMCA4 may be trafficked to sperm through a similar mechanism as in mice [19]. Human oEVs also contain proteins important for sperm function including endothelial nitric oxide synthase (eNOS) and PMCA1 [19] as identified by western blot analysis of oEVs isolated from Fallopian tube luminal fluid [19]. eNOS encodes NOS3 and has been shown to be important for male fertility [62]. PMCA1 also regulates Ca^2+^ and compensates for the lack of PMCA4 in *Pmca4*^−/−^ male mice [19]. The presence of PMCA1, PMCA4, and eNOS in human Fallopian tube oEVs suggests that these oEV protein cargos are conserved in mammals and may therefore modulate similar processes for mammalian reproductive functions [19].

Ferraz and colleagues examined the protein content of oEVs isolated from dogs and cats at various stages of the reproductive cycle [25,28]. oEVs from dogs contained protein cargo relating to sperm function including testis-specific gene antigen 10 (TSGA10), cystein-rich secretory protein-3 (CRISP3), and CRISP2 [25]. TSGA10 and CRISP3 have both been associated with increased sperm motility in mice and horses, respectively [63,64]. CRISP2 is present in the sperm acrosome and tail, and can bind the cation channel sperm-associated protein 1 (CatSper) [65]. Additionally, sperm motility is disrupted in *Crisp2*^−/−^ mice [65], implicating CRISP2 as a required protein for sperm function. The addition of domestic counterpart oEVs from dogs and cats prevented premature acrosomal reaction in vitro for red wolves and cheetahs, respectively [25]. The addition of oEVs from dogs improved sperm motility in red wolves. However, the addition of domestic cat oEVs did not change the motility of cheetah sperm. These findings suggest that, in dogs and cats, the effect of oEVs on sperm function may be modulated in a species-specific manner.

The oEVs from domestic cats contain protein cargo that is important for sperm function and fertilization, as oEVs directly interact with sperm in vitro and improve both sperm motility and fertilization rate [28]. These oEVs were shown to directly interact with the outer acrosomal membrane, sperm head and midpiece (Figure 3D). Proteomic analysis also revealed that these oEVs from cats contained proteins involved in sperm motility (ATP1A4, ATP2B4, DNAJA1, APOB, CCDC39, CCDC40, DPCD, DNAH1, DNAH5, NPHP4, PGK2, PLTP, TEKT2, and TEKT3), sperm-egg recognition (NME5, NME7, NME9, NME1,CAD, MTOR, and UPRT), sperm-ZP binding (CCT2, CCT3, CCT4, CCT5, CCT7, CCT8, OVGP1, and TCP1), and metabolism (ATP synthase, V-type proton ATPase subunit E, ATP binding, plasma membrane calcium-transporting ATPase 2, ADP/ATP translocase 1, mitochondrial, ATP synthase, cytochrome c oxidase, acyl-CoA dehydrogenase, succinate-CoA ligase (ADP/GDP-forming) subunit alpha, NADPH, ATPase Na^+^/K^+^ transporting, and ATPase plasma membrane Ca^2+^ transporting). Common proteins found in oEVs in other species that are important for sperm viability and fertilization, such as HSP70 and OVGP1, were also identified in oEVs isolated from cats [28]. The addition of oEVs increased the percentage of motile sperm in vitro by about 10–15% compared to sperm without oEVs, with this trend lasting 24 h. However, progressive motility of the sperm was not affected and hyperactivation was only increased for 1 h after oEV treatment [28]. The addition of oEVs also improved sperm integrity as it prevented sperm from prematurely undergoing the acrosomal reaction in non-capacitation conditions in vitro. oEV incubation also improved fertilization rate by 23%. Contrary to oEV studies in pigs, addition of oEVs has no effect on polyspermy rates in cats.

Polyspermy is prevalent in porcine IVF compared to other species. Polyspermy in pigs can be seen in up to 90% of IVF fertilized oocytes while the prevalence is less than 5% in vivo [66]. Polyspermy causes incorrect number and partitioning of chromosomes in the resulting embryo and leads to an unsuccessful pregnancy. Similar to mice, dogs, and cats, oEVs isolated from pigs can be incorporated into the sperm head, midpiece, as well as the flagellum (Figure 3E) [59]. Alcântara-Neto and colleagues demonstrated that the addition of porcine oEVs to the culture medium reduces polyspermy in vitro [26]. Additionally, porcine oEVs contain several proteins related to reproductive physiology and exosome secretion including MYH9, OVGP1, ANXA1, ANXA4, ANXA5, RPS5, valosin containing protein (VCP), FLOT1, CLU, TSG101, and HSPA1A. Annexin proteins have been described as sperm binding proteins in bulls. ANXA1, ANXA4, and ANXA5 are located on the cell surface of the oviduct epithelium, particularly in ciliated cells, where bull sperm most often bind. Incubation of sperm with antibodies against annexin proteins blocks sperm binding in the oviduct and ANXA5 in particular has been shown to bind dead or apoptotic sperm [67]. Based on these findings, it was hypothesized that (1) OVGP1 and MYH9 from the oEVs bind to the ZP, and subsequently lead to the modification of carbohydrate and protein composition, causing ZP hardening and preventing polyspermy, and that (2) annexin proteins from porcine oEVs bind to the sperm, prevent the arrival of mass number of sperm at the oocyte, and contribute to improved IVF efficiency [26,59].

In turtles, high oEV (identified by CD63-positive particles) concentration is detected during months that correlate with sperm storage (January, September and December) [27]. In contrast, oEV concentration is low during the breeding season (May). During breeding season, exosomes were found close to ciliated cells, as opposed to dispersed throughout the oviductal lumen [27]. Previous studies have shown that oEV molecular cargo plays a role in sperm function, viability and fertilization in the oviduct. Therefore, this increase in oEV concentration during sperm storage may contribute to maintaining sperm viability in turtle oviducts.

In hens, exosomes (also identified by CD63-positive particles) were detected in the sperm storage tubules (SSTs) of the oviduct. Huang and colleagues showed that exosomes found in the hen reproductive tract were secreted in response to sperm and affected sperm viability [68]. After artificial insemination, expression of CD63-positive particles (exosomes) in the SSTs is decreased but was increased in the lumen of STTs. This finding suggests that the presence of sperm causes the secretion of oEVs to aid in sperm viability and storage. The addition of exosomes isolated from the vagina, but not those isolated from the utero-vaginal junction (UVJ) significantly reduced sperm viability (from about 70% to 35%) in vitro. Also, the addition of UVJ exosomes had no significant effect on sperm viability. The authors hypothesized that the vagina secretes cytotoxic factors that contribute to sperm selectivity, as only about 1% of total sperm reach the SSTs [69], which would explain why the addition of vaginal exosomes decreased sperm viability [68]. These data in hens indicate that the effects of exosomes on sperm function are tissue specific.

These studies provide evidence that suggest that the binding of these oEVs to sperm supplies proteins that aid in essential processes for fertilization including sperm function, sperm motility, sperm viability, and sperm–egg interactions. However, further studies using genetic ablations of the target molecules in vivo or pharmacological inhibition in vitro are required to functionally address whether these protein cargos isolated from the oEVs have physiological meaning to sperm function.

### 4.3. Effect of oEVs on Embryos

**Figure 4:**
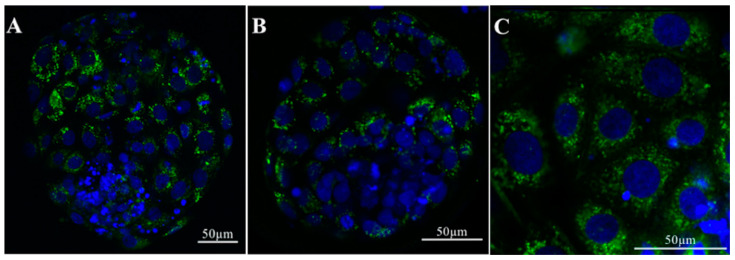
Incorporation of bovine oEVs into the blastocyst. (**A**–**C**) oEVs from bovine oviducts labeled with green fluorescent dye (PKH67) are incorporated into cytoplasmic contents in the bovine blastocyst. (**C**) High magnification image shows that in vivo-derived oEVs attach to the blastocyst plasma membrane and are internalized into the cytoplasm. Hoechst 3342 staining; nucleus. Reprint with permission from [24].

In vitro culture environment has been shown to drastically affect transcript levels of several genes related to important embryonic processes including metabolism, growth, immune and epigenetic factors, apoptosis, and preimplantation embryonic development [70]. It is well-accepted that the presence of the oviductal fluid has positive effects on embryo development. However, it is still unclear whether this positive effect can be attributed to the presence of oEVs. Almiñana and colleagues showed that, in cows, in vivo-derived EVs from the oviduct were able to pass through the ZP and can be taken up into the cytoplasmic content in blastocysts (Figure 4) [24]. Green fluorescent labeled EVs were detected in the cytosolic component of the embryos after 18–20 h of incubation [24]. These data suggest that molecular cargos in oEVs can be incorporated into the embryos.

In vivo-derived bovine oEVs enhance embryo development and quality [24]. The addition of frozen oEVs upregulated several genes involved in embryonic development and viability in the embryos [71]. Further studies have shown that oEVs collected from the ampulla and isthmus can affect embryonic development differently. In cows, embryos incubated with oEVs collected from the isthmus had a blastocyst yield of 91.3% compared to a 62.2% when incubated with oEVs from the ampulla [7]. Supplementation with isthmic oEVs increased aquaporin 3 (*AQP3*) expression in cultured embryos, which could potentially augment cryotolerance of bovine embryos [7]. Coincubation with isthmic oEVs also increased expression of DNA methyltransferase 3A (*DNMT3A*) and the imprinted gene, small nuclear ribonucleoprotein polypeptide N (*SNRPN*), in embryos. Aberrant methylation patterns have been shown to lead to imprinting disorders, suggesting that oEVs in the isthmus could contribute to the regulation of normal methylation patterns in embryos [7]. Moreover, it was previously shown that blastocysts cultured in synthetic oviductal fluid (SOF) supplemented with isthmic oEVs had a higher survival rate (80.1%) compared to blastocysts from SOF with added fetal calf serum (34.5%) and blastocysts from SOF with no additions (50.5%) [72]. However, further investigation is required to determine the exact function of proteins derived from isthmic oEVs on embryo development in vivo.

Incubation of frozen oEVs increases genes involved in the embryo developmental process in bovine embryos [71]. One of the genes increased in embryos is Y box binding protein 1 (*YBX1*). *YBX1* has been shown to have implications in maternal mRNA stability as well as early embryonic development in zebrafish [73]. The addition of frozen oEVs also led to the downregulation of 96 genes, some of which are targetable by miRNAs present in the oEVs. Some of these genes include Sp3 transcription factor (*SP3*), which is involved in trophectoderm cell differentiation, *NANOG*, a homeobox transcription factor that promotes pluripotency and regulates early embryogenesis, and toll-like receptor (TLR) pathways (TLR2-10) that aid in the innate immune response. Sirtuin 1 (*SIRT1*, a regulator of *NANOG* expression) as well as many apoptosis-promoting genes (such as *APAF1*, *TRIM32*, *MAP3K1*, and *ATF2*) were also downregulated in bovine embryos when treated with frozen oEVs. These data suggest that the addition of oEVs to culture media causes changes in the embryo transcriptome including the repression of apoptosis and promotion of embryonic development and viability.

Nevertheless, there have been contrasting results regarding how freezing oEVs affects oEV integrity. Furthermore, there have been conflicting findings on how frozen and fresh oEVs affect embryos in culture. Some studies have shown that freezing does not affect the characteristic of EVs [74,75] whereas others suggest that it might [76,77,78]. As mentioned above, different isolation procedures could alter the characteristics of EVs. Therefore, systemic studies comparing sizes, concentrations, and most importantly the molecular cargo, from fresh vs. frozen oEVs need to be performed.

In mice, the presence of sperm in the reproductive tract and exogenous gonadotropins affect the number and concentration of oEVs, which subsequently impact embryo development. The addition of oEVs derived from donor oviductal fluid in culture media was shown to improve the quality of embryos more so than embryos cultured with oEVs derived from recipient oviductal fluid [79]. The addition of murine oEVs at 1.87 × 10^11^ particles/mL also reduced reactive oxygen species (ROS) [80]. Interestingly, the proportion of oEVs in the oviductal fluid was higher for donors (1.29 × 10^9^ ± 6.32 × 10^7^ particles/mL) than the recipients (5.90 × 10^8^ ± 5.29 × 10^6^). Moreover, the protein concentration was also higher in oEVs isolated from the donors (356.3 ± 28.3 µg/mL) compared to those from the recipients (175.7 ± 44.7 µg/mL). The differences observed in the oEVs between the two groups were hypothesized to be due to the presence of sperm in the donor females, as well as the fact that these donor females were superovulated, neither of which occurred in the recipients. While the study showed that the addition of oEVs improved embryo quality, a decrease in blastocyst development rate was observed when ammonium levels exceeded 300 µM. These high ammonium concentration levels were observed in cultures where oEVs were pooled from 20 animals. Specifically, there was a significant decrease in blastocyst development in the cultures where oEVs from donor fluid was pooled from 10 vs. 20 mice (88.4% vs. 76.2%, respectively) [79]. Ammonium levels of over 300 µM significantly decreased the development of the inner cell mass, and inhibited the ability of embryos to regulate intracellular pH [81]. This finding indicates that the appropriate amount of oEVs is necessary to maintain optimal conditions for preimplantation embryo development.

In cats, the co-incubation of cat oEVs improved fertilization rate (by 23%) as well as increased the number of eight-cell embryos (12%) and blastocysts (8%) produced in vitro [28]. This suggests that oEVs provide important factors that promote preimplantation embryonic development in mammalian species [28].

Previous studies have shown that production of embryos in vitro creates higher DNA methylation pattern compared to embryos produced in vivo [82,83,84]. Supplementation with oviductal fluid in in vitro culture changes the DNA methylation pattern in bovine embryos. Similarly, co-culture of embryos with oEVs also reduces 5-methylcytosine (5-MC) levels and improves overall blastocyst rates in mice [80]. In cows, to determine how the oviductal fluid collected at different developmental stages impacts the methylation status at the CpG islands of genes associated with embryonic development, embryos were co-cultured with oviductal fluid isolated at the 1–16-cell, 1–8-cell, or the 8–16-cell stages (referred to as OF1-16, OF1-8, or OF8-16, respectively) [85]. These genes included mitochondrial transcription termination factor 2 (*MTERF2*) [86], ATP-binding cassette 7 (*ABCA7*; expressed on sperm and is important for fertilization) [87], olfactomedin 1 (*OLFM1*; important for neuronal growth and implantation) [88], and GDP-Mannose 4,6-Dehydratase (*GMDS*; important for brain development) [89]. Treatment with OF1-16, OF1-8, or OF8-16 decreased methylation levels of *MTERF2* (20.0, 26.2, and 32.9, respectively) compared to a group without oviductal fluid (56.2%). Methylation of *ABCA7* was only decreased in embryos treated with OF1-16 (31.1%) compared to 65.8% in group without oviductal fluid. OF1-8 increased methylation levels of *OLFM1* (47.1%) in embryos compared to the other treatment groups (OF1-16: 19.4%, OF8-16: 29.4%) and no oviductal fluid (24.1%). *MTERF2* transcript was increased in each OF treatment group whereas long interspersed nuclear element-1 (*LINE1*) expression decreased when treated with OF1-16. This finding indicates that the oviductal fluid (potentially also oEVs) present at various chronological processes during embryo development differentially impact DNA methylation patterning in the resulting embryos in vitro [85].

These studies have shown that oEVs directly interact with embryos and therefore are likely responsible for the regulation of the embryo transcriptome. Furthermore, these data provide evidence that in vitro culture affects the embryo transcriptome and the addition of oviductal fluid and oEVs improve embryo quality, preimplantation embryo development, expression of important genes, and DNA methylation patterning.

## 5. Are oEVs the Missing Key in Assisted Reproductive Technologies?

### 5.1. Natural Conception vs. In Vitro Fertilization

Assisted reproductive technologies (ARTs) include in vitro fertilization (IVF), gamete intrafallopian transfer (GIFT), zygote intrafallopian transfer (ZIFT), intrauterine insemination (IUI), and embryo transfer (ET). In the United States, according to the Center for Disease Control and Prevention’s report for 2017, approximately 306,197 ART cycles were performed, resulting in 73,831 live births out of 3.79 million [90]. Approximately 1.9% of infants born in the United States are conceived using ARTs. IVF procedures include the development of the embryo to the blastocyst stage in culture media, or for 3–7 days, depending on the protocol, followed by the transfer of embryo(s) into the uterine cavity. With these procedures, the entire Fallopian tube is bypassed. In procedures like ZIFT and GIFT, the zygote or gametes are transferred directly into the Fallopian tube. While there are no differences between implantation rates using ZIFT compared to IVF in healthy women, pregnancy and implantation rates are significantly higher when ZIFT is performed in women with repeated implantation failure using IVF (35.1% success rate with ZIFT vs 11.1% for IVF) [91]. It is obvious that the Fallopian tube is superior for fertilization and embryo development than artificially modified conditions in vitro. However, it is virtually unknown how oEVs from the Fallopian tube provide an optimal microenvironment for gametes and embryos in humans.

### 5.2. oEVs Could Improve the Quality of ART-Derived Embryos

Within the Fallopian tube, embryos are bathed in fluid containing oEVs, which are missing in culture media. Although the majority of babies born using ARTs are healthy, culture conditions in ARTs have been associated with epigenetic changes in the embryo. Alteration of expression patterns in imprinted genes may indicate imprinting disorders. Out of 10,000 live births following ARTs procedures, 3.9 children are diagnosed with Angelman syndrome, 3.9 with Beckwith-Wiedemann syndrome, 2.2 with Prader–Willi syndrome, and 1.5 with Silver-Russel syndrome [92]. The frequency of these imprinting disorders in the normal population is approximately 2 children out of every 10,000 live births [92].

As mentioned above, co-culture of embryos with oviductal fluid collected at various stages of embryo development differentially altered methylation patterns in bovine embryos [85]. Blastocysts that were cultured in SOF with addition of fetal calf serum exhibited downregulation of the imprinted gene called small nuclear ribonucleoprotein polypeptide N (*SNRPN*) compared to blastocysts cultured in synthetic fluid containing oEVs [72]. Accordingly, decreased *SNRPN* expression has been associated with Prader–Willi syndrome [93]. Therefore, it is possible that in vivo-derived oEVs are directly involved in methylation control in embryos. However, to date, we still cannot pin-point as to which proteins or molecular cargos from oEVs are responsible for normal embryo development. It is likely that the cumulation of distinct proteins present chronologically in the oEVs is correspondingly responsible for proper functions of gametes and embryos at various developmental stages.

## 6. Conclusions

Here, we have demonstrated that oviductal fluid, and more specifically, oEVs are important for embryo quality. oEVs have a role in regulating gamete functions and interactions as well as embryos in the oviduct in order to support fertilization and preimplantation embryo development. oEVs within the oviductal fluid are also important for supporting a proper microenvironment within the oviduct. Therefore, these data reviewed in this article suggest that the addition of oEVs to in vitro culture conditions could help to better mimic the oviductal environment and aid in higher quality embryo production, as well as the reduction of polyspermy.

## Figures and Tables

**Table 1:** Sizes of extracellular vesicles in the oviduct in different species.

Species	Size Distribution of oEVs	References
Mouse	oEVs: 25–100 nm	[23]
Human	Exosomes: <100 nm; Microvesicles: 0.1–1 µm	[19]
Cow	Exosomes: 30–100 nm; Microvesicles: 100–250 nm	[24]
Dog	oEVs: 158.9 ± 33.73 nm	[25]
Pig	Exosomes: 30–150 nm; Microvesicles: >150 nm	[26]
Turtle	oEVs: 50–130 nm	[27]
Cat	oEVs: 40–150 nm	[28]
